# What is the role of *Ewingella americana* in humans? A case report in a healthy 4-year-old girl

**DOI:** 10.1186/s12879-019-4021-4

**Published:** 2019-05-06

**Authors:** Susanna Esposito, Francesco Miconi, Daniela Molinari, Emanuela Savarese, Federica Celi, Livio Marchese, Sara Valloscuro, Giovanni Miconi, Nicola Principi

**Affiliations:** 1 Pediatric Clinic, Department of Surgical and Biomedical Sciences, Università degli Studi di Perugia, Piazza Menghini 1, 06129 Perugia, Italy; 2 Pediatric Clinic, Azienda Ospedaliera di Terni, Terni, Italy; 3 Laboratory Unit, Azienda Ospedaliera di Terni, Terni, Italy; 4 Università degli Studi di Milano, Milan, Italy

**Keywords:** Antibiotics, Antinfective therapy, Bacteremic pneumonia, Case report, Community-acquired pneumonia, *Ewingella Americana*

## Abstract

**Background:**

*Ewingella americana* (*Ea*) is a Gram-negative, lactose-fermenting, oxidase-negative and catalase-positive bacterium that was first described in 1983 as a new genus and species in the family *Enterobacteriaceae*. It is not known whether *Ea* is a true pathogen or simply an opportunistic infectious agent, as most of the cases have been described in patients at risk.

**Case presentation:**

A 4-year-old girl described here was hospitalized due to a productive cough over the previous 3 weeks and a fever > 38 °C associated with tachypnea over the previous 2 days. Her familial and personal medical histories were negative for relevant diseases, including respiratory infections. At admission, she was febrile (axillary temperature 39.2 °C) and had dyspnea with retractions, grunting and nasal flaring. A chest examination revealed fine crackling rales in the left upper field associated with bilateral wheezing. A chest X-ray revealed segmental consolidation of the lingula of the left lung. Laboratory tests revealed leukocytosis (15.,800 white blood cells/mm^3^ with 50.3% neutrophils), a slight increase in serum C-reactive protein (11.9 mg/L) and normal procalcitonin values (< 0.12 ng/mL). A nasopharyngeal swab culture did not reveal viral or bacterial respiratory pathogens, including atypical bacteria. A blood culture revealed the presence of a Gram-negative, lactose-fermenting rod that was oxidase negative and catalase positive. The isolate was identified by means of the VITEK®2 identification system (bioMérieux, Firenze, Italy) as *Ea*. This identification was confirmed by sequencing the 16 s ribosomal deoxyribonucleic acid (rDNA). The pathogen was sensitive to aminoglycoside, fluoroquinolones, carbapenems, cefotaxime, and ceftazidime but was intermediate against sulfametoxazole/trimethoprim and resistant to amoxicillin-clavulanic acid, fosfomycin, and oxacillin. The child was immediately treated orally with amoxicillin-clavulanic acid and erythromycin. Based on the results of a blood culture and sensitivity tests, the amoxicillin-clavulanic acid medication was stopped after 3 days. Erythromycin was continued for a total of 10 days, and the child was discharged after 3 days in the hospital. Follow-up visit 1 month later did not reveal any respiratory problems.

**Conclusion:**

This case shows that *Ea* infections in healthy subjects are mild even in pediatric age, and the need for antibiotic therapy is debated. Cases occurring in subjects with underlying chronic disease can be significantly more complicated and require appropriate antibiotic therapy.

## Background

*Ewingella americana* (*Ea*) is a Gram-negative, lactose-fermenting, oxidase-negative and catalase-positive bacterium that was first described by Grimont et al. in 1983 as a new genus and species in the family *Enterobacteriaceae* [1]. *Ea* is very common in some vegetables and mushrooms, in which it can cause a browning disorder called internal stipe necrosis [2]. However, in humans, the detection of *Ea* in the tissues and bodily fluids of patients with infectious diseases is very uncommon, and the true role of this agent has not been precisely defined. Clinical infections due to *Ea* have been reported to cause peritonitis, conjunctivitis, bacteremia, and pneumonia [3–6]. Colonization in wound and sputum were also reported in patients without causing clinical infection [3, 7]. Sepsis and even death from Waterhouse-Friderichsen syndrome due to *Ea* has also been reported [8]. *Ea* is seen in adut patients who were immunosuppressed due to diabetes mellitus, bone marrow transplantation, chemotherapy, end stage renal disease, and use of mercaptopurine [5, 6]. However, though some cases were published in the literature, little is known about the natural habitat of *Ea*. As source of infection some authors proposed the citrate solutions prepared in the hospital for coagulations study, the domestic water, the contamination of ice bath, the contamination of catheter or inadequate hand hygiene [9].

*Ea* can survive in water and grow preferentially at 4 °C and this isolate is not known to be the normal flora of respiratory tract [9]. This paper reports a case of community-acquired bacteremic pneumonia due to *Ea* in an otherwise healthy 4-year-old girl that can be useful for increasing our knowledge regarding the clinical relevance of *Ea*.

## Case presentation

The 4-year-old girl described here was hospitalized in the Pediatric Ward of the General Hospital of Terni in the Umbria Region of Italy due to a productive cough over the previous 3 weeks and a fever > 38 °C associated with tachypnea over the previous 2 days. Her familial and personal medical histories were negative for relevant diseases, including respiratory infections. The child was born at term after a normal pregnancy. At admission, her physical and neurodevelopmental growth were in the normal range. However, she was febrile (axillary temperature 39.2 °C) and had dyspnea with retractions, grunting and nasal flaring. An examination revealed a respiratory rate of 40 breaths/min, heart rate of 111 beats/min, oxygen saturation in room air of 96%, and blood pressure of 104/68 mmHg. A chest examination revealed fine crackling rales in the left upper field associated with bilateral wheezing. All other systems were normal.

Chest radiography and a series of laboratory tests, including a blood culture, complete blood cell count, serum C-reactive protein (CRP) and procalcitonin levels, liver enzymes, renal function markers, and *Chlamydophila pneumoniae* and *Mycoplasma pneumoniae* antibody concentrations were performed. Moreover, a nasopharyngeal swab for the identification of respiratory bacteria and a Mantoux test were performed. A chest X-ray revealed segmental consolidation of the lingula of the left lung. Laboratory tests revealed leukocytosis (15,800 white blood cells/mm^3^ with 50.3% neutrophils), a slight increase in serum C-reactive protein (11.9 mg/L) and normal procalcitonin values (< 0.12 ng/mL). Blood liver enzymes and renal function markers were normal. A nasopharyngeal swab culture did not reveal viral or bacterial respiratory pathogens, including atypical bacteria. The Mantoux test was negative after 48 h, and atypical bacterial antibodies were not detected.

A blood culture revealed the presence of a Gram-negative, lactose-fermenting rod that was oxidase negative and catalase positive. The isolate was identified by means of the VITEK®2 identification system (bioMérieux, Firenze, Italy) as *Ea*. This identification was confirmed by sequencing the 16 s ribosomal deoxyribonucleic acid (rDNA) and comparing the result with a previously published genetic sequence. The antimicrobial susceptibility test carried out by disk diffusion method showed susceptibilities against amikacin, ampicillin, cefotaxime, cefoxitin, ceftazidime, ciprofloxacin, gentamicin, imipenem, piperacillin, piperacillin-tazobactam, and tetracycline, but was intermediate against sulfametoxazole/trimethoprim and resistant to amoxicillin-clavulanic acid, fosfomycin, and oxacillin.

Table 1 summarizes clinical, laboratory and radiological findings at admission, during hospitalization and at the follow-up visit. The child was immediately treated orally with amoxicillin-clavulanic acid (50 mg/kg/die of the amoxicillin component in 3 doses) and erythromycin (50 mg/kg/die in 3 divided doses). Moreover, aerosolized beclometasone in saline solution was administered two times per day. The improvement in the patient’s respiratory condition was rapid. Two days after hospital admission, the resolution of her respiratory abnormalities was complete, CRP levels returned to normal values, and the aerosolized therapy was stopped. Moreover, based on the results of a blood culture and sensitivity tests, the amoxicillin-clavulanic acid medication was stopped after 3 days. Erythromycin was continued for a total of 10 days, and the child was discharged after 3 days in the hospital. Follow-up visit 1 month later did not reveal any respiratory problems.

**Table 1:** Clinical, laboratory and radiological findings in a 4-year-old girl with community-acquired bacteremic pneumonia

Findings	Admission	After 2 days	After 4 days (at discharge)	After 28 days (follow-up)
Clinical objectivity	Productive cough, fever, tachypnea, dyspnea with retractions, grunting, nasal flaring, fine crackling rales in the left upper field, bilateral wheezing	Resolution of dyspnea, improvement of the rales	Good cardio-respiratory objectivity, no more rales at auscultation	Good general conditions and complete resolution of symptoms
Oxygen saturation	96%	98%	99%	99%
Chest X-ray	Segmental consolidation of the lingula in the left lung			
Leukocytes (cells/mm^3^)	15,810	10,340	8600	
Neutrophils, %	50.3	50.8	45	
Lymphocytes, %	39.3	31.5	40	
Monocytes, %	7.9	9.9	9	
Eosinophils, %	2.1	7.5	5	
Basophils, %	0.4	0.3	1	
Procalcitonin, ng/mL	< 0.12			
C reactive protein, mg/dL	1.19	1.71	0.31	
IgG anti-*Chlamydia pneumoniae*	0.2			
IgM anti-*Chlamydia pneumoniae*	0.7			
IgG anti-*Mycoplasma pneumoniae*	< 0.1			
IgG anti-*Mycoplasma pneumoniae*	2.4			
Blood culture	Positive for *Ewingella americana*			
Mantoux Test	Negative			

Management of the case was approved by the Ethics Committee of General Hospital of Terni, Italy (2018-PED-02). The patients’ parents provided their written informed consent for the management of their child and the publication of the case report.

## Discussion and conclusion

*Ea* has been isolated from a variety of clinical specimens, including blood, wound swabs and sputum. Most patients with it are old and/or are suffering from severe underlying conditions, such as complicated surgeries [10], injuries from accidents [11], drug abuse [12], and renal failure [13]. Some patients have had diabetes, received immunosuppressive therapy, were HIV positive, or suffered from other chronic infections [14]. Cases in otherwise healthy subjects are very rare. This is why *Ea* can be considered an opportunistic bacterium. On the other hand, cases with severe clinical manifestations, including sepsis, have been almost exclusively reported in subjects who, due to their age or the presence of an underlying acute or chronic severe clinical condition, could be considered at risk of severe superimposed bacterial infections.

In healthy subjects, *Ea* infections are generally mild and limited to conjunctivitis and, as in the case reported here, respiratory infections with rapid favorable outcomes. Death has been described in only two cases, both in patients with conditions favoring infectious disease development, such as being at an older age and having severe traumatic injuries. The first case was that of a previously healthy old woman who suffered from Waterhouse-Friderichsen syndrome [8], while the second case occurred in a 30-year-old man who developed nosocomial pneumonia following admission to the hospital due to a road traffic accident [11]. However, because of their underlying conditions, in this second case it was difficult to determine whether the *Ea* infection was indeed the cause of death [11]. Interestingly, *Ea* infections are even rarer in children than in adults. A case of sepsis in an infant with congenital nephropathy [15] and a case of acute conjunctivitis [16] have been described. This is the first case of CAP due to *Ea* documented in an otherwise healthy child.

The clinical course of this child seems to indicate that in otherwise healthy subjects *Ea* infections are mild and can resolve spontaneously in few days even when antibiotic treatment is not effective in vitro. In this case, antibiotic therapy was chosen according to the recommendations of several official guidelines that indicate that, in hospitalized children ≥4 years old with radiographically documented CAP, the addition of a macrolide to traditional beta-lactam therapy is an acceptable solution when an atypical bacterial etiology cannot be excluded [17]. At admission, blood culture results and serum *Chlamydophyla pneumoniae* and *Mycoplasma pneumoniae* antibody levels were not available. Moreover, both the blood neutrophil count and acute phase reactant serum levels were compatible with a potential atypical bacterial etiology. As the etiology could not be determined, combined therapy with amoxicillin-clavulanic acid and erythromycin was prescribed. However, when the results of antibiotic sensitivity tests became available, it was clear that amoxicillin-clavulanic acid could not be effective. In contrast, erythromycin, despite not being evaluated against this patient’s pathogen, is generally poorly effective against all *Enterobacteriaceae*, including *Ea* [18]. Consequently, it seems unlikely that the very rapid favorable evolution of this case of CAP could be based exclusively on the antibiotic treatments.

However, the development of an *Ea* infection in patients at risk can pose difficult therapeutic problems. In these cases, antibiotics remain mandatory, but the choice is not simple. Usually, *Ea* is considered resistant to several β-lactamases, mainly first- and second-generation cephalosporins, but sensitive to third- and fourth-generation drugs from the same group [18]. Penicillins have variable sensitivity, which explains why phenotypes with ampicillin and amoxicillin resistance seem to be inconsistent in the literature [18]. Aminoglycosides, fluoroquinolones, and sulfametoxazole/trimethoprim are generally effective. However, cases of multidrug-resistant *Ea* have been described in which there was a failure of the initially prescribed antibiotic therapy. Pound et al. described a case of *Ea* CAP in a 77-year-old female already suffering from a *Mycobacterium tuberculosis* infection and Crohn’s disease; in this case, *Ea* was sensitive only to ticarcillin/clavulanate, cefotetan, and SMX/TMP, and the correct therapy was prescribed only after the results of a sputum culture and microbial sensitivity tests were available [14].

In conclusion, this case shows that *Ea* infections in healthy subjects are mild even in pediatric age, and the need for antibiotic therapy is debated. Cases occurring in subjects with underlying chronic disease can be significantly more complicated and require antibiotic therapy. The choice of the right antimicrobial can be made difficult by the emergence of resistance to first-line drugs.

## Abbreviations

CAP: Community-acquired pneumonia
*Ea*: *Ewingella americana*
rDNA: Ribosomal deoxyribonucleic acid

## References

[1] Grimont PA, Farmer JJ, Grimont F, Asbury MA, Brenner DJ, Deval C (1983). *Ewingella americana* gen.Nov., sp.nov., a new *Enterobacteriaceae* isolated from clinical specimens. Ann Microbiol (Paris).

[2] Inglis PW, Peberdy JF (1996). Isolation of *Ewingella americana* from the cultivated mushroom, *Agaricus bisporus*. Current Microbiol.

[3] Ryoo NH, Ha JS, Jeon DS, Kim JR, Kim HC (2005). A case of pneumonia caused by *Ewingella americana* in a patient with chronic renal failure. J Korean Med Sci.

[4] Da Costa PS, Tostes MM, de Carvalho Valle LM (2000). A case of keratoconjunctivitis due to *Ewingella americana* and a review of unusual organisms causing external eye infections. Braz J Infect Dis.

[5] Kati C, Bibashi E, Kokolina E, Sofianou D (1999). Case of peritonitis caused by *Ewingella americana* in a patient undergoing continuous ambulatory peritoneal dialysis. J Clin Microbiol.

[6] Lartigue Marie-Frédérique, Nordmann Patrice, Edelstein Mikhail V., Cuzon Gaëlle, Brisse Sylvain, Poirel Laurent (2013). Characterization of an extended-spectrum class A β-lactamase from a novel enterobacterial species taxonomically related to Rahnella spp./Ewingella spp. Journal of Antimicrobial Chemotherapy.

[7] Bear N, Klugman KP, Tobiansky L, Koornhof HJ (1986). Wound colonization by *Ewingella americana*. J Clin Microbiol.

[8] Tsokos Michael (2003). Fatal Waterhouse-Friderichsen Syndrome due to Ewingella americana Infection. The American Journal of Forensic Medicine and Pathology.

[9] McNeil MM, Davis BJ, Solomon SL, Anderson RL, Shulman ST, Gardner S, Kabat K, Martone WJ (1987). *Ewingella americana*: recurrent pseudobacteremia from a persistent environmental reservoir. J Clin Microbiol.

[10] Pien FD, Bruce AE (1986). Nosocomial *Ewingella americana* bacteremia in an intensive care unit. Arch Intern Med.

[11] Bukhari SZ, Hussain WM, Fatani MI, Ashshi AM (2008). Multi-drug resistant *Ewingella americana*. Saudi Medical J.

[12] Hassan S, Amer S, Mittal C, Sharma R (2012). *Ewingella americana*: an emerging true pathogen. Case Rep Infect Dis.

[13] Li L, Shen J, Tao J, Xue Z (2014). Peritonitis caused by *Ewingella americana* in a patient with peritoneal dialysis: a case report. J Med Case Rep.

[14] Pound MW, Tart SB, Okoye O (2007). Multidrug-resistant *Ewingella americana*: a case report and review of the literature. Ann Pharmacother.

[15] Cobos Carrascosa E, Daza Torres A, Campos Aguilera A, Giménez Sánchez F (2014). Sepsis by *Ewingella americana* in an infant with congenital nephropathy. An Pediatr (Barc).

[16] Maraki S (2012). Acute conjunctivitis caused by *Ewingella americana*. J Pediatr Ophthalmol Strabismus.

[17] Esposito S, Cohen R, Domingo JD, Greenberg D, Heininger U, Pecurariu OF (2012). Antibiotic therapy for pediatric community-acquired pneumonia: do we know when, what and for how long to treat?. Pediatr Infect Dis J.

[18] Stock I, Sherwood KJ, Wiedemann B (2003). Natural antibiotic susceptibility of *Ewingella americana* strains. J Chemother.

